# Feasibility of axicabtagene ciloleucel in the outpatient setting: primary analysis of prospective trial

**DOI:** 10.1038/s41409-025-02551-z

**Published:** 2025-03-24

**Authors:** Bhagirathbhai Dholaria, Shakthi T. Bhaskar, Vivek G. Patel, Eden Biltibo, Reena Jayani, Salyka Sengsayadeth, Andrew Jallouk, James Jerkins, Brittney Baer, Ali Nur, Muhamed Baljevic, Bipin N. Savani, David Morgan, Adetola A. Kassim, Olalekan O. Oluwole

**Affiliations:** Department of Hematology-Oncology and Stem Cell Transplantation, Vanderbilt University Medical Center, Nashville, TN USA

**Keywords:** B-cell lymphoma, Immunotherapy

## Abstract

The pivotal trials of axicabtagene ciloleucel (axi-cel) chimeric Antigen receptor (CAR-T) therapy and other CD19 CAR-Ts were mainly done in the inpatient setting. We conducted a single-center non-randomized open label prospective clinical trial (NCT05108805). Objective was to evaluate the feasibility and safety of outpatient administration of axi-cel for relapsed/refractory diffuse B cell lymphoma (R/R DLBCL) using continuous remote patient monitoring with wearable devices and telemedicine. We enrolled 25 and treated 20 patients on this clinical trial from November 2021 to October 2023. The median follow up was 374 days (95% CI: 364–484). The median age was 69 years and 18 of 20 patients (90%) received prophylactic corticosteroids. Nineteen patients got admitted for management of cytokine release syndrome (CRS) in the first 4 weeks. The median duration of hospital stay was 5.5 days (range: 0–21). Two patients required ICU stay due to pre-existing conditions exacerbated by CRS. Grade 2 CRS was seen in 40% and no pt had grade ≥3 CRS. Grade 3 Immune effector cell-associated neurotoxicity syndrome (ICANS) was seen in 20% and none had grade ≥4 ICANS. No treatment-related death by last follow-up. The results of our pilot study confirm the feasibility of outpatient administration of axi-cel.

## Introduction

Chimeric antigen receptor T-cell (CAR-T) therapy has led to durable responses in the treatment of lymphoid malignancies and its use is projected to increase, expanding to other malignancies and even autoimmune conditions [1–3]. However, CAR-T therapy is associated with serious side- effects including cytokine release syndrome (CRS) and Immune effector cell-associated neurotoxicity syndrome (ICANS) which requires close monitoring and need for rapid interventions such as tocilizumab and/or steroids. Given these reasons, the pivotal trials of CD19 CAR T therapy were mainly done in the inpatient setting [4–7]. The data gathered in the inpatient setting were used to develop toxicity management protocols [8, 9]. Through multiple iterations, these protocols provided effective means to intervene early to shorten the duration and prevent higher grade CRS or ICANS [8–10]. Early use of tocilizumab and steroid prophylaxis have led to a more tolerable toxicity profile for CAR-T in general, it has also facilitated access to CAR T therapy from select academic centers to community practices [11]. However, the inpatient location for the delivery of CAR-T remained the standard which has contributed in no small way to the high cost of CAR-T treatment [12, 13]. As the adoption of CAR-T grows, so will hospital usage and the poorer quality of life of patients in the inpatient compared to outpatient setting will remain a concern [14]. Axicabtagene ciloleucel (axi-cel) have the highest objective responses of the class and manufacturing reliability which have led to its widespread use [1, 15–17]. Though CRS and ICANS are class effects of CAR-T, there is significant variability in the incidence and severity among CAR T products with axi-cel having highest incidence of severe CRS and ICANS [18]. Retrospective single-center studies have been published showing feasibility of outpatient administration of various CD19 CAR T products, but well-designed prospective studies are lacking [11, 19]. A recent phase 2 study showed safety and feasibility of outpatient administration of lisocabtagene maraleucel (liso-cel) at community cancer centers which has much lower risk for high grade CRS and ICANS compared to axi-cel [20].

## Methods

We designed a prospective single center clinical trial to administer and monitor axi-cel in the outpatient setting. The primary objective was the feasibility of administration and monitoring in the outpatient setting. “feasibility” refers to whether outpatient axi-cel can be safely and practically administered, assessing key aspects like recruitment rates, adherence to study procedures, data collection methods, and potential side effects. Secondary objectives were to identify risk factors that preclude outpatient administration and to assess the impact of telemedicine and remote monitoring on specific outcomes including CRS and ICANS. Progression-free survival (PFS) was calculated using Kaplan- Meier estimates from the day of axi-cel infusion (day 0) to disease progression, last follow up or death from any cause. Similarly, overall survival (OS) was calculated from day 0 to last follow up or death from any cause. Statistical analysis was done using NCSS version 12.0 (NCSS, LLC^®^, Utah, USA).

Patients age ≥ 18 years with relapsed/refractory diffuse B cell lymphoma (R/R DLBCL) who met the US Food and Drug Administration (FDA) package label for axi-cel were enrolled on the study. We required all patients to have a reliable caregiver for the treatment process and reside within a 30-mile radius of study site for 4 weeks following axi-cel infusion. We educated the patient and caregiver in relevant aspects of the CAR-T and documented competence with a pre-test and post-test (supplementary appendix). We also performed two screening procedures, one at study entry prior to leukapheresis and the second prior to lymphodepletion therapy to ensure that they met eligibility for the outpatient administration. Those who were ineligible for outpatient therapy received axi-cel in the inpatient setting. Patients who remained eligible to proceed with outpatient therapy received lymphodepleting chemotherapy and axi-cel infusion in the outpatient clinic (remained open daily regardless of weekend or holidays) and they were subsequently monitored with daily clinic visit till day 14. They also had two telemedicine visits (4 pm and 10 pm) during which review of system questions were asked and ICE score calculated. Patients wore the remote monitoring (Biofourmis^©^) devices through 14 days from axi-cel infusion for continuous monitoring of skin temperature, heart rate and oxygen saturation [21]. Blood pressure cuff was applied at specified intervals and data uploaded automatically using a cell phone interphase [21]. The vital signs were available for the treatment team to see in real time via remote monitoring portal. To optimize safety, we implemented early intervention for toxicity management in all patients and prophylactic corticosteroids for high-risk patients according to ZUMA-1 cohort 4 and 6, respectively [10, 22]. Bridging therapy was allowed at discretion of treating physician. Patients were admitted if they developed high grade fever, suspicion for Grade ≥2 CRS or any grade ICANS. Inpatient admission for any other organ toxicity was allowed at the discretion of the treating physician. Patients were discharged from outpatient CAR T clinic around day 30 to return home.

## Results

From January 2022 to October 2023, we screened 36 patients, 25 underwent leukapheresis on study and 20 received axi-cel infusion which were included in the primary analysis (supplementary fig. 1). The data cut off for primary analysis was September 12, 2024, at the median follow up was 374 days (95% CI: 364–484).

The median patient age was 69 years (range: 35–81). At time of study screening, Ann Arbor stage III-IV disease in 18 (90%) of patients and elevated lactate dehydrogenase (LDH) in 13 (65%) were reported. One patient had history of central nervous system (CNS) involvement with DLBCL. The median prior lines of therapies were 2(range: 1–3) with 4 (20%) patients received ≥ 3 prior therapies before enrollment. No patient had prior autologous hematopoietic cell transplantation before enrollment. Median time from consent to axi-cel infusion (day 0) was 42 (range: 6–74) days. A total of 18 (90%) patients were high risk (Ann Arbor stage III / IV and / or high tumor burden evidenced by high LDH) and received prophylactic dexamethasone 10 mg/day, days 0–2 per FDA package label. Bridging therapy was given to 6 (30%) patients. The most common agents used for bridging were polatuzumab vedotin with rituximab (*N* = 3), followed by dexamethasone (*N* = 2) and radiation therapy (*N* = 1).

### Safety

A total of 18 (90%) of patients remained outpatient at least 72 h after axi-cel administration, and one (5%) patient remained outpatient through day 30. The median time from day 0 to hospitalization was 3 (range: 1–6) days and the median hospital length of stay was 5.5 (range 0–21) days. The reason for hospitalization was CRS in all admitted patients, and the median duration of CRS and ICANS was 3 days each. No patient developed grade ≥3 CRS or grade ≥4 ICANS. Two patients required ICU stay prior to day+30, one for exacerbation of pre-existing COPD and the other patient had history of heart disease developed arrhythmia during CRS. There were no treatment-related deaths in first 180 days from axi-cel infusion. Five patients who lived within 30 miles of our clinic were able to stay at their home. The rest of the patients stayed in hotels or lodges near clinic. Five patients died by the last follow up (disease related- 4, bacterial sepsis- 1). Table 1 provides detailed outcomes.

**Table 1:** Outcomes of the patients.

Outcomes	*N* = 20 (%)
Day 30 responses	
CR	14 (70%)
SD	4 (20%)
PD	2 (10%)
Day 90 responses (*N* = 17)	
CR	11 (65%)
PR	1 (6%)
SD	2 (12%)
PD	3 (18%)
Best response	
CR	17 (85%)
SD	1 (5%)
PD	2 (10%)
Median Time to hospitalization from day 0 (*N* = 19), days	3 (1–6)
Median inpatient days by D + 30, days	5.5 (0–21)
Pts with ICU admission through D + 30	2 (10%)
CRS	
Grade 0	1 (5%)
Grade 1	11 (55%)
Grade 2	8 (40%)
Median time to CRS from day 0, days	3 (1–6)
Median duration of CRS, days	3 (1–5)
ICANS	
Grade 0	12 (60%)
Grade 1	4 (20%)
Grade 3	4 (20%)
Median time to ICANS from day 0, days	5 (4–10)
Median duration of ICANS, days	3 (1–9)
Additional Steroids given	8 (40%)
Tocilizumab given	17 (85%)
Median doses of tocilizumab	2 (0–3)

*CRS* cytokine release syndrome, *ICANS* Immune effector cell-associated neurotoxicity syndrome, *CR* complete remission, *PR* partial remission, *SD* stable disease, *PD* progressive disease.

### Efficacy

**Fig. 1:**
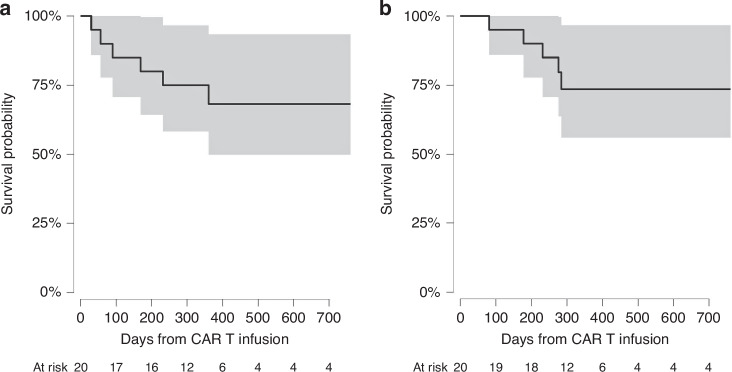
Patient outcomes. Progression-free (**a**) and overall survival (**b**).

Five patients developed progressive/refractory disease at the last follow-up with four deaths from progressive disease. Among patients who achieved complete remission on day 30 after axi-cel (*N* = 14), cumulative incidence of relapse was 19% (95% CI: 5%–67%) at one-year (supplementary Fig. 2). The median PFS or OS was not reached by last follow up. The 1-year estimated PFS was 68% (95% CI: 47%–90%) and OS was 74% (95% CI: 54%–94%) (Fig. 1).

## Discussion

The results of this prospective study showed that axi-cel despite having high rate of CRS and ICANS can be safely administered in the outpatient setting without compromising safety and efficacy outcomes of patients with R/R DLBCL. Our study outcomes were favorable compared to the pivotal ZUMA 1 study of axi-cel. Whereas ZUMA-1 reported grade 3 or higher CRS and ICANS in 13% and 28% of patients, our cohort had 0% and 20% respectively. Furthermore, the median duration of hospitalization in ZUMA-1 was 15 days compared to 5.5 days in current study [1]. This suggests that starting CAR-T in the outpatient setting and admitting those who required hospitalization can lead to fewer hospital days used in total and judging by the cost of an inpatient bed (CAR-T needs a monitored or step-down level of care), the cost savings can be significant.

There are few possible reasons why the safety profile in our study is better than previously published data for axi-cel [1]. First, disease burden prior to start of lymphodepletion was lower in our study given routine use of bridging therapy. Our method of using telemedicine for visits played a key role in our ability to provide close monitoring of patients and capture early CRS and ICANS events which resulted in early utilization of tocilizumab and steroids. The fact that patients performed the telemedicine in their home or apartment led to improved quality of life. Secondly, our use of prophylactic corticosteroids in high-risk patients and early intervention in all patients per cohort 4 and 6 of ZUMA 1 [10, 22] ensured that most CRS and ICANS were grade 1–2 and whenever a higher grade developed, the duration was short which positively impacted our length of stay to 5 days compared to 15 in the pivotal study. Thirdly, we provided specific education to patient and caregivers and verified competence to understand specific questions related to the review of systems, reliably measure vital signs, operate a device such as tablet or smart phone. This not only equipped the patient and family with essential tools to partner with us in the care process, and we hope to expand this to other aspects of our care delivery process. There are specific requirements that we believe are essential for a successful outpatient CAR-T process including an extended hours outpatient clinic open 7 days a week, ability to directly admit patients without going through the Emergency Department and a 24-hour contact person on call. Some of these were previously published by our group [23].

We set out to test whether axi-cel, the most efficacious CAR-T in its class, can be safely administered and patients monitored in the outpatient setting using tools such as patient and caregiver education, close monitoring with telemedicine, prompt admission per specified criteria and early intervention for toxicity management. Our study showed that axi-cel can be administered safely in the outpatient setting with careful patient monitoring with a safety profile that is probably better than historical inpatient data and one-third less total hospital length of stay which translates to healthcare savings. Larger studies are needed to confirm our findings in other healthcare settings and disease histologies.

## Supplementary information

supplementary material

Appendix A

## Data Availability

For original data, please contact Bhagirathbhai.r.dholaria@vumc.org
