# Supplementary material for: Feasibility of axicabtagene ciloleucel in the outpatient setting: primary analysis of prospective trial
Source: Bone Marrow Transplant. 2025 Mar 24;60(6):769–72. doi: 10.1038/s41409-025-02551-z (PMC12151859; doi:10.1038/s41409-025-02551-z)

Supplementary figure 1: CONSORT diagram

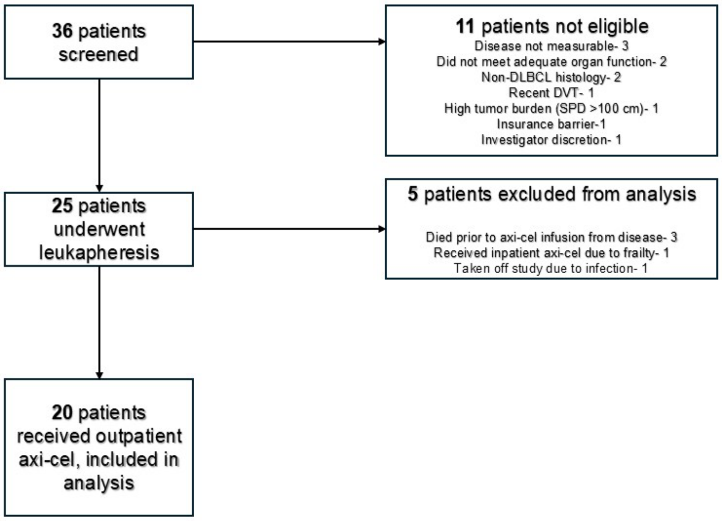

Supplementary figure 2: cumulative incidence of relapse (A) and duration of response (B)

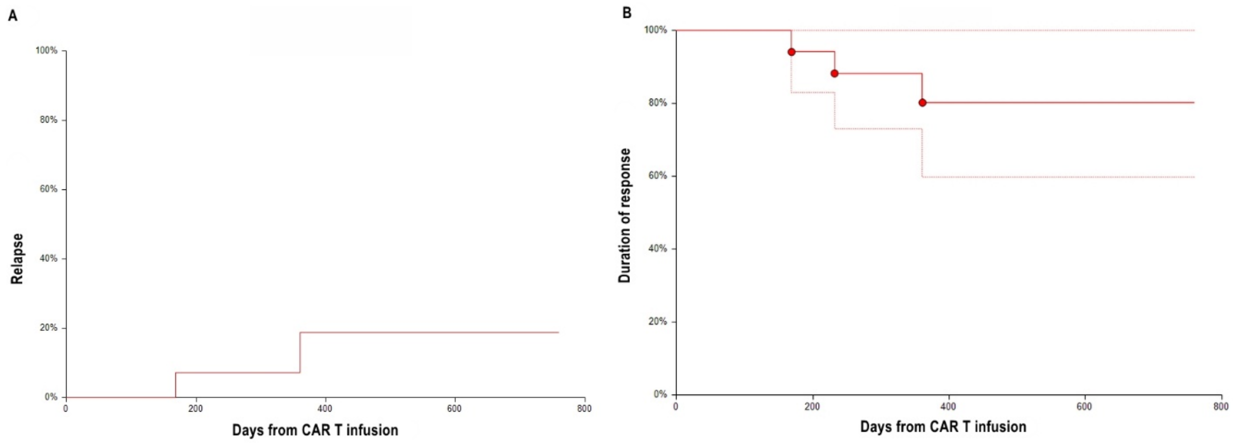

Supplement: Supplementary file 1 — supplementary material [file 41409_2025_2551_MOESM1_ESM.pdf]
