# Supplementary material for: Feasibility of axicabtagene ciloleucel in the outpatient setting: primary analysis of prospective trial
Source: Bone Marrow Transplant. 2025 Mar 24;60(6):769–72. doi: 10.1038/s41409-025-02551-z (PMC12151859; doi:10.1038/s41409-025-02551-z)
Supplement: Supplementary file 2 — Appendix A [file 41409_2025_2551_MOESM2_ESM.pdf]

Appendix A

Vanderbilt University Medical Center
YESCARTA Patient and Caregiver Education: Self-monitoring for CAR T toxicities

Date:

Clinic contact details:
By Phone: CART-T phone number: (615) 497-6865, 24-hour VICC Access Center: (615) 936-8422

General consideration:
You are part of patient's care team and we need your help to monitor the patient's wellbeing while not in the clinic or hospital. It is very important that you pay attention to any new symptoms or signs and report to us immediately. Below is the summary common symptoms/signs of CAR T therapy associated toxicities. This is NOT a complete list and you should not hesitate to call us with any new issue which may arise while at home.

I. Vital Signs: equipment, testing, results

- What equipment will you use?
  - Blood Pressure Cuff – used to take patient's blood pressure and heart rate
  - Pulse Oximeter – used to measure patient's oxygen and heart rate
  - Thermometer – used to take patient's temperature
- How often will take vitals for patient
  - Patient will have vital signs done during clinic visits at **8am**
  - The caregiver will be responsible for vital signs at **6am, 12pm, 4:30, 10pm,**
- How will you report vital signs?
  - Patient will have a video conference call with night NP at **4:30pm and 10:00 PM** ± 1 hour for at least first 14 days after Car-T infusion
  - Patient will call night NP at 6am to report **6am** vital signs.

Things to monitor at Home:

II. Signs of Infection

- Fever: You will take your temperature **6am, 12pm, ≤10pm**,
  - A temperature higher than 100.3 F is a reason to call at any time
- Redness and/or tenderness around patient's venous catheter site
- Dizziness, shortness of breath, cough, altered mental status

III. Cytokine Release Syndrome (CRS)

- What is CRS?
  - This is an inflammatory process that impacts many organs. CRS typically occurs within first week after CAR-T infusion.
- What are CRS symptoms?
  - Fever
  - Chills
  - Dizziness/lightheadedness
  - Shortness of breath
  - Altered mental status
  - Low blood pressure (Please call if systolic blood pressure drops below ____ mm Hg)

    - Fast heart rate (Please call if hear rate is above 120 beats/min)
    - Low blood oxygen levels (Please call if pulse oxygen level fall below 90 %).
- How do we treat CRS?
    - Intravenous fluids
    - Supplemental Oxygen
    - Tocilizumab (medication to reverse CRS)
    - Steroids.

IV. Neurotoxicity

- What is Neurotoxicity?
    - This is a collection of symptoms that impact patient's mental status related to CAR T therapy administration.
- What are Neurotoxicity symptoms?
    - Confusion
    - Headache
    - Delirium (confused thinking and reduced awareness of environment, for example to time and place; usually with rapid onset)
    - Difficulty finding words/speaking
    - Difficulty following commands
    - Memory lapses
    - Hallucinations (seeing or hearing things which does not exist)
    - Dizziness
    - Tremors
    - Seizures/ convulsions
    - Lethargy (lack of energy and enthusiasm).
- How do we treat Neurotoxicity?
    - Steroids.

V. Monitoring

- How will the patient be monitored?
    - You will be seen every day in our Outpatient Transplant Unit (OTU) at 8:00am and a nurse will visit the apartment at 4:30pm ± 1 hour daily for 14 days after your CAR-T infusion (Day 0)
    - You will have your labs and vitals taken daily in clinic.
    - You will have a video conference telehealth visit with the night NP at 10pm. At that time, you will take your vital signs and answer questions.
    - You will take your vitals at 6am and then have a morning phone call with night NP to report overnight vital signs.
    - Once you are discharged from the OTU, you will transition to the CAR-T long term care clinic. This typically occurs between 14 and 30 days after CAR-T infusion.

Official Use only

Version date:	Approved by:

Appendix B

Vanderbilt University Medical Center
YESCARTA Caregiver Pre-Test

Date: **Subject ID:**

1. What temperature is considered serious and warrants a call to Vanderbilt?
    a. 100.0°F
    b. 99.5°F
    c. 100.4°F
    d. 97.6°F
2. Which symptom may mean the patient is experiencing neurotoxicity?
    a. Tremors
    b. Confusion
    c. Being extra sleepy
    d. All of the above
3. What times do we need the patient to connect to the telehealth monitoring system?
    a. 10:00 PM
    b. 2:00 AM
    c. 6:00 AM
    d. None of the above
4. Please perform 3 blood pressures on patient and write down values:
    a.
    b.
    c.
5. Please take the patient's temperature 3 times and write down values:
    a.
    b.
    c.
6. Please take patient's pulse oxygen levels and write down values:
    a.
    b.
    c.
7. Please check patient's heart rate and write down values:
    a.
    b.
    c.
8. What symptom may mean the patient is experiencing cytokine release syndrome (CRS)?
    a. Chills
    b. Low blood pressure
    c. Fever
    d. All of the above

Official Use only

Appendix Version Date:	Score:	Pass/Fail	Comment:	Reviewer's initials:

Appendix C

Vanderbilt University Medical Center
YESCARTA Caregiver Post-Test

Date: **Subject ID:**

1. What temperature is considered serious and warrants a call to Vanderbilt?
   a. 100.0°F
   b. 99.5°F
   c. 100.4°F
   d. 97.6°F
2. Which symptom may mean the patient is experiencing neurotoxicity?
   a. Tremors
   b. Confusion
   c. Being extra sleepy
   d. All of the above
3. What times do we need the patient to connect to the telehealth monitoring system?
   a. 10:00 PM
   b. 2:00 AM
   c. 6:00 AM
   d. None of the above
4. Please perform 3 blood pressures on patient and write down values:
   a.
   b.
   c.
5. Please take the patient's temperature 3 times and write down values:
   a.
   b.
   c.
6. Please take patient's pulse oxygen levels and write down values:
   a.
   b.
   c.
7. Please check patient's heart rate and write down values:
   a.
   b.
   c.
8. What symptom may mean the patient is experiencing cytokine release syndrome (CRS)?
   a. Chills
   b. Low blood pressure
   c. Fever
   d. All of the above

Official Use only

Score	Pass/Fail	Comment:	Reviewer's initials:
